# Assessing the Impact of Copy Number Variants on miRNA Genes in Autism by Monte Carlo Simulation

**DOI:** 10.1371/journal.pone.0090947

**Published:** 2014-03-25

**Authors:** Maurizio Marrale, Nadia Ninfa Albanese, Francesco Calì, Valentino Romano

**Affiliations:** 1 Dipartimento di Fisica e Chimica, Università di Palermo, Palermo, Italy; 2 U.O.C. di Genetica Medica Laboratorio di Genetica Molecolare, Associazione Oasi Maria SS. (I.R.C.C.S.), Troina, Italy; Seoul National University College of Medicine, Korea, Republic of

## Abstract

Autism Spectrum Disorders (ASDs) are childhood neurodevelopmental disorders with complex genetic origins. Previous studies have investigated the role of *de novo* Copy Number Variants (CNVs) and microRNAs as important but distinct etiological factors in ASD. We developed a novel computational procedure to assess the potential pathogenic role of microRNA genes overlapping *de novo* CNVs in ASD patients. Here we show that for chromosomes # 1, 2 and 22 the actual number of miRNA loci affected by *de novo* CNVs in patients was found significantly higher than that estimated by Monte Carlo simulation of random CNV events. Out of 24 miRNA genes over-represented in CNVs from these three chromosomes only hsa-mir-4436b-1 and hsa-mir-4436b-2 have not been detected in CNVs from non-autistic subjects as reported in the Database of Genomic Variants. Altogether the results reported in this study represent a first step towards a full understanding of how a dysregulated expression of the 24 miRNAs genes affect neurodevelopment in autism. We also propose that the procedure used in this study can be effectively applied to CNVs/miRNA genes association data in other genomic disorders beyond autism.

## Introduction

The Autism Spectrum Disorders (ASDs, MIM: 209850) are a heterogeneous group of childhood diseases characterized by abnormalities in social behaviour and communication, as well as patterns of restricted and repetitive behaviors [1]. The spectrum of autism reflects dimensional variability of each core symptom as well as the occurrence of co-morbid conditions (intellectual disability, epilepsy, dysmorphisms etc.). Like an epidemic, in part due to improved diagnostic tools, the global prevalence of autism and other pervasive developmental disorders has increased over the years reaching the current, impressive, figure of 1/160 children [2]. Twin studies have demonstrated a much higher concordance rates for the disease in monozygotic twins (92%) than in dizygotic twins (10%) [3], [4], indicating a strong genetic basis for autism susceptibility, also supported by the presence of autistic features in several monogenic disorders (e.g., Fragile X syndrome, Tuberous sclerosis). However, despite these progresses the identity of genetic factors still remains unknown in the majority of patients and it is likely, that overall, the causes of autism are more complex than previously thought involving an interaction of genetic, epigenetic and environmental factors all interfering with the normal course of neurodevelopment [5]–[7].

In 2007 Copy Number Variants (CNVs) were for the first time recognized as important genetic factors in ASD [8]. CNVs are a class of inherited or *de novo* genomic mutations duplicating (“Gains”) or deleting (“Losses”) DNA segments 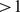 Kb, thus altering the normal dosage of the overlapping genes. According to their frequency they can be divided into unique, rare (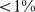) or recurrent. Since 2007, many studies have investigated CNVs in autism, to assess their functional impact, the biological networks where the genes in these CNVs are involved and the general burden of CNVs in these individuals (*e.g.* see references [9]–[11]). Extended, multiplex families are more likely to carry heritable risk factors while, in contrast, sporadic ASD families have demonstrated a higher rate of *de novo* CNVs [8], [12]. A striking observation was the high prevalence of *de novo* CNVs in both sporadic and familial cases of ASD compared with controls. Rare, *de novo* or inherited CNVs were observed in 5–10% of idiopathic ASD cases.

Studies performed so far have highlighted the pathogenic role of CNVs in terms of dosage change for protein-coding genes [10], [13]–[15], without taking into account the potential involvement of non-coding RNA, particularly microRNAs genes (see reference [16] for a recent exception).

miRNAs are an important class of post-trascriptional regulators each governing the expression of tens or even hundreds of proteins in both differentiated cells and during development. miRNA transcripts undergo several processing steps occurring first in the nucleus, then in the cytoplasm where the mature 22–25 nucleotide-long miRNA [17], [18] enacts mRNA translational repression or cleavage by binding to the 3′-untranslated region of their respective target mRNAs [19]. Only a few studies have investigated the ASD transcriptome in *post-mortem* brain samples, so far [20]–[22]. Other studies have used mRNA from lymphoblastoid cell lines or peripheral blood cells isolated from patients [23]–[26]. Disruption of miRNA expression has been repeatedly reported in several microarray studies and believed to be linked to pathogenesis in autism [27]–[31]. However, lymphoblastoid cell lines are not the best proxy for neural tissue and only very few miRNAs displayed consistent dysregulation among different studies and patients.

In this paper, we aim to expand our knowledge on the pathogenic role of miRNA in autism by investigating the associations between *de novo* CNVs and miRNA genes. For this reason we developed a novel and powerful computational procedure based on Monte Carlo randomization [32] and applied it to several published *de novo* CNV datasets from patients. Our positive findings consist in the identification of 24 miRNA genes over-represented in *de novo* CNV from chromosomes nos. 1, 2 and 22 and therefore likely to play a pathogenic role in autism.

## Results

### Study design and preliminary analyses

**Figure 1:**
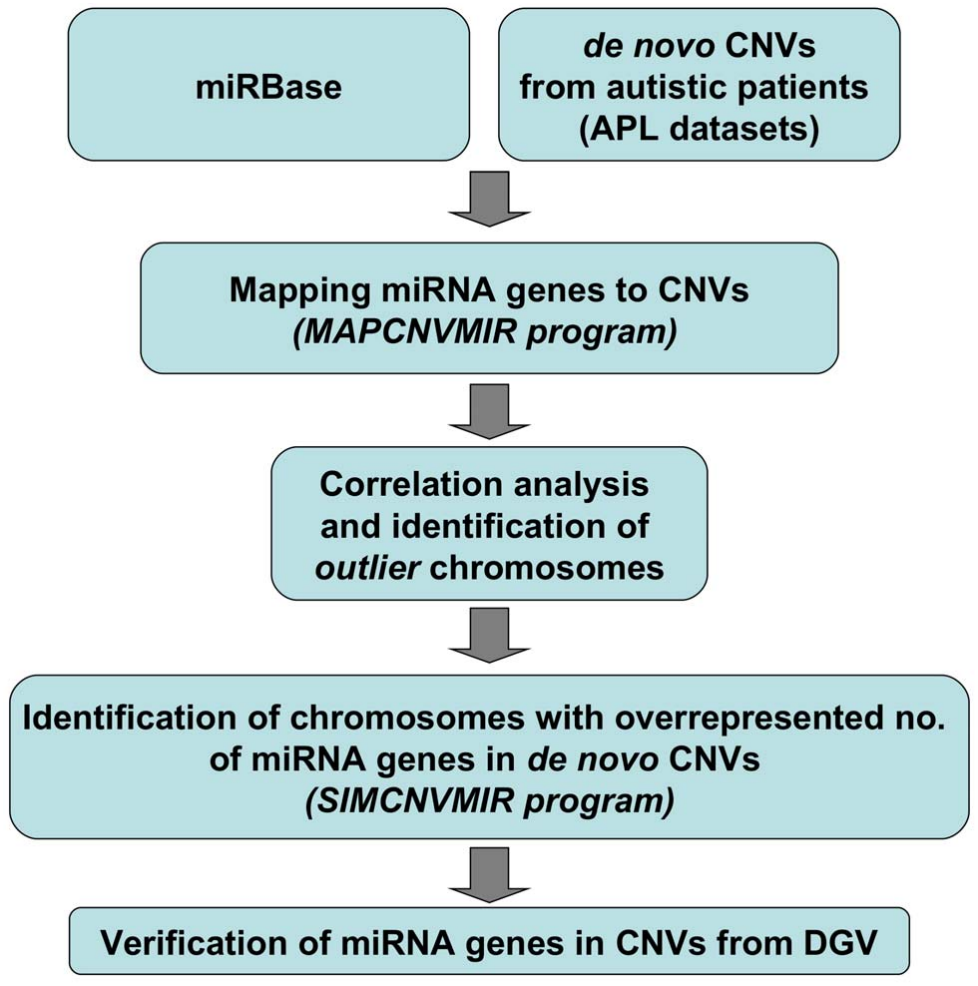
Overview of this study and source data. In this study we have used previously published data from 192 autistic patients (the “APL datasets” of Table S1) bearing overall 178 *de novo* CNVs (118 CNV_Losses and 60 CNV_Gains) with unique start and end positions.

The general strategy and steps of our study are outlined in the flow-chart of Figure 1. We developed the MAPCNVMIR programme to map all human miRNA genes from miRBase to 178 *de novo* CNVs from 192 autistic patients (“APL datasets”, see Table S1). Only 64 out of 178 CNVs were found to overlap at least one miRNA gene. In addition, 145 miRNA genes were identified within distinct or partly overlapping *de novo* CNVs (Table S1) spread over 20 chromosomes. No miRNA genes included in *de novo* CNVs were detected in chromosomes # 11, 13, 14, Y.

**Figure 2:**
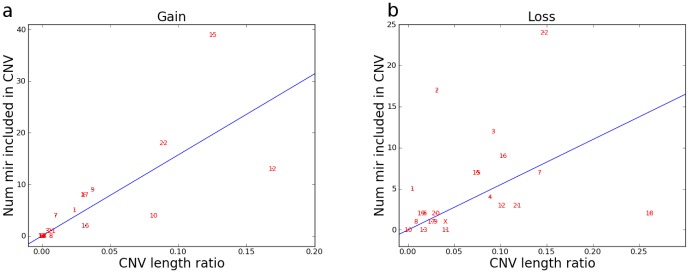
Correlation graph between the no. of miRNA genes in *de novo* CNVs and the CNV/Chr. lengths ratio (Ratio #2) For each chromosome, the number of miRNA genes associated to CNVs is plotted as a function of the fractional length of CNV over the chromosome's size for Gains (a) and Losses (b), respectively. The graphs show that whereas the majority of data points lay very close to the best-fit line, indicating that the two variables are positively correlated, few chromosomes instead behave as outliers in which certain CNVs appear to affect a no. of miRNA genes higher than expected (data used for the graphs were taken from Table 1).

For each chromosome we compared the number and the length of all human microRNA genes reported so far in miRBase with the length of the chromosome. The fractional length of miRNA genes over the size of the chromosome (R_1_ ratios) are of the order of 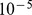 for all chromosomes except for chromosome 19 (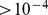) (see Table 1). This result shows that the fraction of the length of the chromosome covered by microRNA genes is very similar for all chromosomes (except chr. 19). On the other hand, the fractional length of CNVs over the size of the chromosome (R_2_ ratios),computed separately for CNV_Gains and CNV_Losses (Table 1) using the MAPCNVMIR software developed by us (see below), largely differ among the various chromosomes, from a value of 0.0006 up to value of 0.2618. This highlights that the fractional length of chromosomes covered by CNVs can be very dissimilar among chromosomes. Indeed, there are several instances (e.g., chr. # 10, 12, 15 and 22 for CNV_Gains and chr. # 7, 12, 16, 18, 21 and 22 for CNV_Losses) of CNVs affecting large regions of a particular chromosome. Therefore, an analysis was performed between the number of miRNA genes overlapping *de novo* CNVs and the R2 ratios in order to find possible correlations. Furthermore, the number of miRNA genes is also variable. Figures 2a and 2b show that, for many chromosomes, the number of miRNA genes included in CNVs follow a linear trend as function of the total length of both CNV_Gains and CNV_Losses. In order to quantify this kind of correlation, values of correlation coefficients were calculated and were found to be larger for CNV_Gains (r = 0.75765) than CNV_Losses (r = 0.32732). This finding is consistent with the larger spread of CNV_Losses compared to CNV_Gains. On the other hand, some chromosomes do not follow the linear trend and are characterized by a large number of miRNA genes associated to CNVs (see Figure 2 and Table 1). For example,chromosome 22 for CNV_Gains and chromosome 2 for CNV_Losses appear as “outliers” since they include a much higher number of miRNA genes in *de novo* CNVs than expected. However, the latter analysis does not allow us to classify the observed dissimilarities according to a statistically significant criterion.

**Table 1:** Fractional lengths of miRNA genes and CNVs in relation to chromosome's size.

*Chromosome*				*Gain*		*Loss*	
*#*	*Length (bp)*	*# of miRNA genes*	*Ratio #1*	*Ratio #2*	*#hits*	*Ratio #2*	*#hits*
1	249250621	127	4.05×10^−5^	0.0241	5	0.0050	5
2	243199373	98	3.10×10^−5^	0.0009	0	0.0314	17
3	198022430	76	3.21×10^−5^	0.0041	1	0.0923	12
4	191154276	56	2.31×10^−5^	*N/A*	*N/A*	0.0886	4
5	180915260	67	3.03×10^−5^	*N/A*	*N/A*	0.0763	7
6	171115067	54	2.56×10^−5^	0.0019	0	0.0187	2
7	159138663	67	3.41×10^−5^	0.0103	4	0.1425	7
8	146364022	70	3.54×10^−5^	0.0068	0	0.0088	1
9	141213431	71	3.95×10^−5^	0.0371	9	0.0297	1
10	135534747	61	3.66×10^−5^	0.0822	4	0.0005	0
11	135006516	69	4.03×10^−5^	0.0002	0	0.0410	0
12	133851895	57	3.48×10^−5^	0.1691	13	0.1013	3
13	115169878	37	2.62×10^−5^	0.0009	0	0.0169	0
14	107349540	88	6.58×10^−5^	0.0006	0	*N/A*	*N/A*
15	102531392	57	4.68×10^−5^	0.1254	39	0.0742	7
16	90354753	48	4.40×10^−5^	0.0320	2	0.1032	9
17	81195210	83	7.89×10^−5^	0.0317	8	0.0256	1
18	78077248	30	2.74×10^−5^	0.0006	0	0.2618	2
19	59128983	108	1.47×10^−4^	*N/A*	*N/A*	0.0145	2
20	63025520	40	5.16×10^−5^	*N/A*	*N/A*	0.0301	2
21	48129895	15	2.65×10^−5^	0.0074	1	0.1183	3
22	51304566	36	5.59×10^−5^	0.0893	18	0.1473	24
X	155270560	108	5.77×10^−5^	0.0304	8	0.0406	1

Ratio #1: The ratio between the sum of lengths of all miRNA genes in a chromosome and the total length of the chromosome; Ratio #2: The ratio between the sum of lengths of all *de novo* CNVs in a chromosome and the total length of the chromosome; # hits: For each chromosome, the total no. of identical and/or different miRNA genes included in all *de novo* CNVs detected in patients.

### Monte Carlo randomization

**Figure 3:**
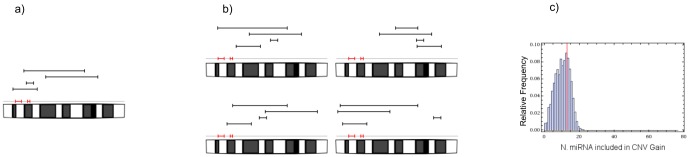
Schematic representation of the counting process of miRNA genes included in *de novo* CNVs of autistic patients. The small black rectangles close to chromosome are the miRNA genes, whereas the various segments above represent the various CNVs within the chromosome. An “hit” is an overlap between a CNV and a miRNA gene. In a) four “hits” are shown. b) Four examples of random distributions of simulated CNVs within the chromosome, keeping fixed the lenght of each CNV and changing its start/end positions. Clockwise from top left, the numbers of “hits” are 2, 0, 6 and 1, respectively. For each chromosome we carried out 10^6^ simulations. c) Finally, the histograms displaying the relative frequency of miRNA genes included in randomly located CNVs (“hits”) are obtained and the comparison between experimental data and computed Monte Carlo distribution is performed. Red lines correspond to the no. of miRNA genes detected in *de novo* CNVs from patients. p-values reported in Table 2 are the areas of the histogram to the right side of the red line. (See also Figure 4).

**Figure 4:**
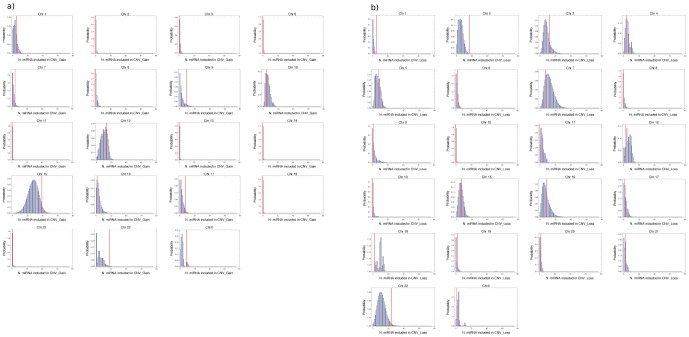
Histograms displaying the relative frequency of miRNA genes included in randomly located CNVs. For each chromosome, the SIMCNVMIR program computes the no. of miRNA genes affected by each randomly distributed CNV realizations and plots the frequency distribution corresponding to 10^6^ realizations. The analyses were performed separately for CNV_Gains (a) and CNV_Losses (b).

In order to assess which “outliers” with a high number of overlaps are actually significantly different from the other chromosomes, we developed the SIMCNVMIR programme which implements a numerical analysis procedure for the identification of all instances (*i.e.*, chromosomes) where the number of miRNA genes overlapping *de novo* CNVs is significantly higher than expected in case of random distribution of (simulated) CNVs. The steps of our computational analysis are reported in Figure 3. The results of this analysis are reported in Figure 4 and Table 2 and show that for chromosomes # 1, 2, and 22 the actual number of miRNA loci affected by *de novo* CNVs in patients is significantly higher (FDR-adjusted p-values 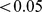) than that estimated by the simulated random CNV events. Specifically, CNV_Gains in chromosome # 22 and CNV_Losses in chromosomes 1 and 2 display an over-representation of microRNA genes (see Table 2 and Figures 4). In Table 2, note that for CNV_Loss in chromosome # 2 the number. of “hits” is higher than the number of distinct miRNA genes (“Unique”), implying that the same miRNA gene is involved. Overall, there are 24 miRNA genes overlapping *de novo* CNVs in the three positive chromosomes (see Table 3). Only two, hsa-mir-4436b-1 and hsa-mir-4436b-2, have not yet been detected in CNVs from the general population (i.e., the DGV database).

**Table 2:** *de novo* CNVs from autistic patients with an overrepresented no. of miRNA genes**.**

chr	GAIN			LOSS		
	Unique	Hits	FDR-adjusted p-value	Unique	Hits	FDR-adjusted p-value
1	5	5	0.45204	5	5	**0.02309**
2	0	0	0.84211	10	17	**0.00449**
3	1	1	0.45642	10	12	0.64896
4	N/A	N/A	N/A	4	4	0.98516
5	N/A	N/A	N/A	7	7	0.73942
6	0	0	0.84211	2	2	0.70911
7	2	4	0.17818	6	7	1
8	0	0	0.84211	1	1	0.70911
9	9	9	0.17818	1	1	0.98516
10	3	4	0.84211	0	0	1
11	0	0	0.84211	0	0	1
12	13	13	0.46568	3	3	1
13	0	0	0.84211	0	0	1
14	0	0	0.84211	N/A	N/A	N/A
15	11	39	0.17818	6	7	0.70911
16	2	2	0.84211	8	9	0.70911
17	8	8	0.13920	1	1	1
18	0	0	0.84211	2	2	1
19	N/A	N/A	N/A	2	2	0.64896
20	N/A	N/A	N/A	2	2	0.64896
21	1	1	0.17818	3	3	0.64896
22	9	18	**0.04349**	9	24	0.07785
X	8	8	0.19599	1	1	1

Unique: the no. of distinct miRNA genes overlapping *de novo* CNVs in patients. Hits: the no. of identical or distinct miRNA genes overlapping *de novo* CNVs in patients. FDR-adjusted p-value: probability of obtaining a number of miRNA genes overlapping randomly-distributed CNVs larger than hits after correction for multiple testing. In bold, chromosomes displaying significant FDR-adjusted p-values (<0.05). N/A: no *de novo* CNVs are present in the APL dataset. For several chromosomes, the no. of unique is smaller than the no. of hits indicating that the same microRNA genes are affected by different *de novo* CNVs.

**Table 3:** List of 24 microRNA genes overrepresented in *de novo* CNVs.

miRNA gene name	Chr	Cytoband	Coordinates (GRCh37)		strand +/−	Size (bp)	Clustered miRNA genes	DGV	no. of Patients	Gain/Loss in Patients	Gain/Loss in positive chr.	Intra/Intergenic miRNA loci
			start	end								
**hsa-mir-2682**	1	1p21.3	98510798	98510907	−	110	hsa-mir-137; hsa-mir-2682.	+	1	Loss	L	none (miRNA gene) none
**hsa-mir-137**	1	1p21.3	98511626	98511727	−	102	hsa-mir-137; hsa-mir-2682.	+	1	Loss	L	(miRNA gene)
**hsa-mir-200b**	1	1p36.33	1102484	1102578	+	95	hsa-mir-200b; hsa-mir-200a; hsa-mir-429.	+	1	Loss	L	Intergenic
**hsa-mir-200a**	1	1p36.33	1103243	1103332	+	90	hsa-mir-200b; hsa-mir-200a; hsa-mir-429.	+	1	Loss	L	Intergenic
**hsa-mir-429**	1	1p36.33	1104385	1104467	+	83	hsa-mir-200b; hsa-mir-200a; hsa-mir-429.	+	1	Loss	L	Intergenic
**hsa-mir-2467**	2	2q37.3	240273419	240273499	−	81	-	+	2	Loss	L	HDAC4 intron 2
**hsa-mir-3133**	2	2q37.3	242417320	242417397	+	78	-	+	2	Loss	L	FARP2 intron 2 - 19
**hsa-mir-4436b-2**	2	2q13	111042430	111042520	+	91	-	−	1	Loss	L	Intergenic
**hsa-mir-4440**	2	2q37.3	239990513	239990610	−	98	-	+	2	Loss	L	HDAC4 intron 1 - 16 - 22
**hsa-mir-4441**	2	2q37.3	240007523	240007622	−	100	-	+	2	Loss	L	HDAC4 intron 5 - 13 - 19
**hsa-mir-4786**	2	2q37.3	240882432	240882511	−	80	-	+	2	Loss	L	NDUFA10 intron 4
**hsa-mir-4267**	2	2q13	110827538	110827619	−	82	-	+	1	Loss	L	Intergenic
**hsa-mir-4436b-1**	2	2q13	110844010	110844100	−	91	-	−	1	Loss	L	MALL intron 1 - 3–4 (antisense)
**hsa-mir-4269**	2	2q37.3	240227157	240227240	+	84	-	+	2	Loss	L	HDAC4 intron 1 - 2
**hsa-mir-149**	2	2q37.3	241395418	241395506	+	89	-	+	2	Loss	L	GPC1 intron 1
**hsa-mir-3618**	22	22q11.21	20073269	20073356	+	88	hsa-mir-3618; hsa-mir-1306.	+	2/3	Gain/Loss	L, G	DGCR8 3′UTR exon 1 - 2
**hsa-mir-1306**	22	22q11.21	20073581	20073665	+	85	hsa-mir-3618; hsa-mir-1306.	+	2/3	Gain/Loss	L, G	DGCR8 3′UTR exon 1 - 2
**hsa-mir-301b**	22	22q11.21	22007270	22007347	+	78	hsa-mir-301b; hsa-mir-130b	+	2	Gain	L, G	PPIL2 intron 1
**hsa-mir-130b**	22	22q11.21	22007593	22007674	+	82	hsa-mir-301b; hsa-mir-130b	+	2	Gain	L, G	PPIL2 exon 2
**hsa-mir-4761**	22	22q11.21	19951276	19951357	+	82	-	+	2/3	Gain/Loss	L, G	COMT 3′UTR exon 1 - 2 - 4 - 5
**hsa-mir-185**	22	22q11.21	20020662	20020743	+	82	-	+	2/3	Gain/Loss	L, G	C22orf25 intron 1 - 2
**hsa-mir-1286**	22	22q11.21	20236657	20236734	−	78	-	+	2/3	Gain/Loss	L, G	RTN4R intron 1 - 2
**hsa-mir-649**	22	22q11.21	21388465	21388561	−	97	-	+	2/3	Gain/Loss	L, G	Intergenic
**hsa-mir-650**	22	22q11.22	23165270	23165365	+	96	-	+	2	Gain	L, G	IGLV2-8 3′UTR exon 1

Information on miRNA gene coordinates, size, gene clustering, intergenic/intragenic loci are as reported in miRBase; no. of patients: number of patients bearing a CNV (including a given miRNA gene), CNVs among patients may have identical or different start/end; Gain/Loss in patients: CNV type overlapping miRNA genes detected in patients (from Table S1); Gain/Loss in positive chr.: CNV type overlapping miRNA genes detected in simulation.

Code scripts of the MAPCNVMIR and SIMCNVMIR programmes are available to readers from the following URL: http://fisicaechimica.unipa.it/cnvmirna/

### Pathway analysis

The two autism-specific miRNA genes (hsa-mir-4436 b-1 and has-mir-4436b-2), are deleted in a patient who bears one CNV of 332,304 bp. Interestingly, they encode the same 3p and 5p mature microRNAs. According to Mirwalk [33], no validated targets are yet known for these two miRNAs. We therefore performed a functional enrichment analysis on the predicted target mRNAs to highlight the significance of these two microRNAs in ASD pathogenesis. The results of this latter analysis, reported in Table 4, show that several pathways identified by miRPath have been already implicated in autism by previous studies, (in bold in Table 4): Lysine degradation [34], Drug metabolism - cytochrome P450 [35], Notch signaling pathway [31], [36], HIF-1 signaling pathway [37], Vasopressin-regulated water reabsorption [38], Natural killer cell mediated cytotoxicity [39]. 2NSD1 [40] and AMT [41] have been previously identified as autism candidate genes.

**Table 4:** KEGG pathways enriched for targets of miRNAs hsa-mir-4436b-3p and -5p identified by mirPath^1^.

	KEGG PATHWAY	p-value	# genes	Target Genes
**hsa-mir-4436b-3p**	**Lysine degradation**	3.30×10^−8^	5	NSD1, KMT2D, PIPOX, KMT2A, KMT2E.
	**Drug metabolism - cytochrome P450**	6.73×10^−6^	1	CYP3A43.
	Other glycan degradation	0.000197	1	NEU1.
	Glycerophospholipid metabolism	0.00029	4	PNPLA7, PGS1, LPCAT3, MBOAT2.
	**Notch signaling pathway**	0.000942	4	CTBP1, APH1A, NOTCH2, MAML1.
	**HIF-1 signaling pathway**	0.006618	5	PGF, EGLN3, PLCG1, ENO3, SLC2A1.
	**Vasopressin-regulated water reabsorption**	0.00847	1	AQP2.
	Glycine, serine and threonine metabolism	0.01361	3	SHMT2, **AMT**, PIPOX.
	Renal cell carcinoma	0.016804	3	PGF, EGLN3, SLC2A1.
	One carbon pool by folate	0.01935	2	SHMT2, **AMT**.
	Pathogenic Escherichia coli infection	0.04271	3	TUBA1A, TUBA8, TUBA1C.
	Graft-versus-host disease	0.04271	3	KIR3DL2, KIR2DL3, KIR3DL1.
	Antigen processing and presentation	0.044381	5	NFYA, NFYB, KIR3DL2, KIR2DL3, KIR3DL1.
	**Natural killer cell mediated cytotoxicity**	0.048633	5	KIR3DL2, KIR2DL3, TNFRSF10B, PLCG1, KIR3DL1.
**hsa-mir-4436b-5p**	Prion diseases	2.16×10^−73^	1	PRNP.
	Sulfur relay system	2.63×10^−9^	1	NFS1.
	Thiamine metabolism	0.002145	1	NFS1.
	**Lysine degradation**	0.008932	1	WHSC1.
	Transcriptional misregulation in cancer	0.023567	2	NCOR1, WHSC1.

1 In bold pathways or genes previouly implicated in autism, see main text.

## Discussion

In recent years, Copy Number Variants and microRNAs have emerged as potentially important etiological factors in ASD. However, until recently, in nearly all the studies, these two topics have been investigated separately in the context of the research of autism. The pathogenic role of CNVs has been interpreted in terms of their effect on the function of the overlapping protein-coding genes. On the other hand, miRNAs have been studied with the aim of uncovering changes in their level of expression in cells isolated from patients (see the Introduction for references) vs. control cells. In our study the focus is on the potential pathogenic role played by miRNA genes/de novo CNVs, instead. For this reason, we developed a new computational procedure implemented in a Fortran-written programme which allows to detect over-representation of miRNA genes in *de novo* CNVs in each chromosome. By this computational analysis based on Monte Carlo simulations we found that in positive chromosomes (FDR-adjusted p values 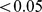, see Table 2 for details) there is a probability of less than 5% to find, by chance, a number of miRNA genes included in CNVs (gain and/or loss) higher than that actually detected in patients. Overall, twenty-four candidate susceptible miRNA genes of autism were identified in our study.

Hereafter, we discuss several potentially critical aspects of this new procedure that may have biased our results and interpretation. Firstly, the results do not appear to be biased by the different distribution of miRNA genes in chromosomes as all chromosomes display very similar miRNA genes length/chromosomes€length ratios. The only exception was chr. 19 which has the highest ratio (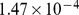), but was not scored positive in a simulation analysis. Secondly, we considered if different ratios between the length of *de novo* CNVs and the length of the chromosome may have accounted for the detection of positive chromosomes. In general, we would expect the number of miRNA genes duplicated or deleted by a CNV to increase linearly with CNV size. However, such a linearity is not always followed, even for chromosomes displaying very similar ratios. Typical examples of this latter situation include CNV_Gains in chromosome 10 (ratio = 0.0822) vs. CNV_Gains in chromosome 22 (ratio = 0.0893) pair consisting of one negative (chr. 10) and one positive (chr. 22) chromosome. Thirdly, we could not perform a simulation analysis, to be used as a negative control, for CNVs detected in individuals from the general population, since the data stored in the Database of Genomic Variants generally refer to blood donors only and not to their parents, thus preventing ascertainment of *de novo* CNVs. However, it is worth mentioning here the results of a recent study by Marcinkowska et al [42], which are consistent with our findings. Indeed, these Authors found that miRNA loci are under-represented in highly polymorphic and well-validated CNVs from the general population (*i.e.*, the Database of Genomic Variants). Fourthly, in Table 1 the absence of “hits” for both CNV_Gain and CNV_Loss for chromosomes # 11, 13, 14, Y is simply due to the lack of autistic patients bearing *de novo* CNVs (Loss or Gain) overlapping miRNA genes (see Table S1). In turn, it can be speculated that, the lack of this type of patient may be ascribed to various factors such as the sample size, the use of low-resolution aCGH platforms, the occurrence of “protective” miRNA loci for autism in these chromosomes. Finally, a more suitable analysis could have involved more homogeneous CNV data from subjects (patients AND unaffected individuals) of the same ethnicity analyzed with aCGH platforms with similar resolution. This was indeed the case for the autistic sample (APL) we have used which was homogenous in relation to ethnicity in that all patients from the APL dataset were “Caucasians” (white north Americans and Europeans). In our study, the use of heterogeneous data concerns instead the different aCGH platforms used (APL and DGV datasets) and the mixed ethnicity of individuals reported in the Database of Genomic Variants. We decided to use such heterogeneous data to increase the chance of collecting a higher number of patients with *de novo* CNVs. This decision had its strengths and drawbacks. For instance, the use of different aCGH platforms may have caused an under-estimation of the number of CNV/miRNA genes associations in positive chromosomes from the APL dataset. In contrast, the use of samples with mixed ethnicity from the DGV database does not seem to have limited the identification of miRNA genes/CNV association in common between DGV and APL datasets. In conclusion, though the use of heterogeneous CNV data may have limited the identification of additional miRNA/CNV associations, it did not prevent the identification of chromosomes with an enrichment of CNVs overlapping miRNA genes.

In our study, the occurrence of the same 22 deleted or duplicated miRNA genes detected in both patients and unaffected individuals (*i.e.*, DGV) strongly suggest that they are low-penetrant risk factors for autism. Difference in penetrance for such duplicated/deleted miRNA genes would be explained by a variety of factors including: (i) prenatal exposure to enviromental risk factors [43], (ii) presence/absence of functional SNPs in susceptibility protein-coding genes of autism [44], (iii) epistasis [45], (iv) epigenetic factors [6], (v) number and type of protein-coding genes co-existing in different CNVs overlapping the same miRNA. It is reassuring that other CNV studies have linked several miRNA genes from this group to autism, and include: hsa-mir-1306, hsa-mir-185, hsa-mir-1286 and hsa-mir-649 genes [16], [46], hsa-mir-200a and hsa-mir-429 [16], [47], hsa-mir-200b and hsa-mir-149 [16]. Furthermore, hsa-miR-185 displays an 1.44-fold upregulation in lymphoblastoid cell lines from autistic patients [48]. Interestingly, this latter finding is consistent with the presence of 3 copies of the hsa-mir-185 gene in the 2 patients from our APL dataset.

To avoid an over-interpretation of our results we have adopted a stringent, conservative criterion according to which we propose that the 22 miRNA genes shared by unaffected individuals and patients should be considered as provisional candidates miRNA genes in ASD. On the other hand, hsa-mir-4436b-1 and hsa-mir-4436b-2, appear at the present time as strong pathogenic candidates in ASD. Unforunately, no validated targets have yet been identified for these two miRNAs. However, functional annotation analysis carried out on predicted mRNA targets for these two miRNAs revealed, that 43% (6/14) of the statistically significant KEGG pathways obtained with hsa-miR-4436b-3p have been already implicated in autism in previous studies (referenced in Table 4), a finding which supports a pathogenic role for this miRNA.

During the preparation of our manuscript, Vaishnavi et al published an article also addressing the impact of microRNAs present in autism-associated Copy Number Variants [16]. Despite, several differences (methodological, type of CNV data used) distinguishing our study from that of Vaishnavi et al., it is worth noting that 8 miRNA genes have been found in common between the two studies (chr. 1: hsa-mir-429, hsa-mir-200a, hsa-mir-200b; chr. 2: hsa-mir-149; chr. 22: hsa-mir-185, hsa-mir-1306, hsa-mir-1286, hsa-mir-649).

## Conclusions

Summing up, positive findings of our study include the identification of 24 miRNA genes over-represented in *de novo* CNVs from 3 chromosomes. Two miRNA genes from this group, hsa-mir-4436b-1 and hsa-mir-4436b-2, are likely to play a significant pathogenic role in autism since they have not been found in CNVs from unaffected individuals. We hope these results will lead experimental research towards a better understanding on the role played by miRNAs in autism. Finally, we propose that the novel procedure used in this study can be effectively applied to CNV/miRNA genes association data from other genomic disorders beyond autism.

## Methods

### Data and databases used

Data on *de novo* CNVs detected in autistic patients were downloaded from three different sources: (i) 71 CNVs from the Autism Chromosome Rearrangements Database [49], [50], (ii) 51 CNVs from Suppl. Table 8 of [10] and (iii) 75 CNVs from Table S1 (document S2) of [13]. Throughout our paper the combined three above sets of data will be named “APL datasets”. CNV (“APL datasets”) data used in this study are reported in the Table S1. CNVs and indels detected in individuals of the general population were downloaded from the Database of Genomic Variants (DGV vers. July 2013) [51]. In our paper this latter dataset is named by the acronym “DGV”. Names, genomic coordinates and chromosomal position for 1,523 human microRNA genes, were obtained from miRBase (vers. 2012). Readers are referred to the article of Griffiths-Jones et al. [52] for an explanation of symbols and nomenclature used for miRNAs and their genes. Genomic coordinates for start and end of CNVs, indels and miRNAs genes were all from Build 37. When necessary, conversion of genomic coordinates between different genome versions was done using the Liftover tool of the UCSC Genome browser [53], [54]. Finally, the list of potentially pathogenic miRNAs was obtained by excluding miRNA genes over-represented in *de novo* CNVs from patients, but not overlapping CNVs from the Database of Genomic Variants (DGV). miRWalk software was used to look for experimentally validated mRNA targets for miRNAs [33]. miRPath [55], [56] was used to identify statistically significant KEGG pathways enriched in the list of predicted miRNA targets (p<0.05; p-values were corrected to account for the False Discovery Rate).

### MAPCNVMIR program (Python)

Data pre-processing included: (i) computation of the total DNA length accounted for by all miRNA genes (L_1_) and CNVs (L_2_) in each chromosome; (ii) computation of R_1_ (R_1_ = L_1_/chromosome length) and R_2_ (R_2_ = L_2_/chromosome length); (iii) counts of the number of miRNA loci overall included in distinct or overlapping CNVs (“hits”). This analysis is performed separately for CNV_Gains and CNV_Losses. Thus, for a given chromosome, “hits” may consist of distinct and/or identical miRNA genes associated to CNVs; on the other hand, we indicate as “unique” the distinct miRNA genes overlapping de novo CNVs in patients.

We developed the MAPCNVMIR programme in Python language to achieve a two-fold task: (i) to calculate for each chromosome the total length of DNA corresponding to the *de novo* CNV regions and (ii) to map the microRNA genes within the *de novo* CNVs detected in patients using their genomic coordinates (Build 37). In order to achieve the first task this programme considers the overlapping DNA regions of different CNVs once only. The programme first initializes for each chromosome an empty array and put the numeric values corresponding to the first (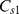) and last (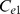) nucleotide positions (“start” and “end”) of the first CNV (of the total list of CNVs reported in Table S1) into a sub-array. Afterwards, the code considers another CNV and compares its initial (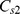) and final (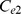) nucleotide positions with those of the first CNV. Three different cases can occur:

1. the first CNV is totally included in the second one (i.e. 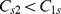 and 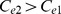) and the values inside the sub-array are replaced by these new ones;
2. the second CNV is partially or totally included in the first one [i.e. (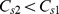 and 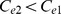) or (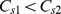 and 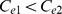) or (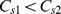 and 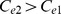)] and the sub-array is composed of the minimum between 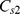 and 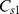 and the maximum between 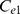 and 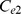;
3. the second CNV does not overlap the first one and in this case a new sub-array with the 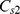 and 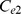 is added to the initial array.

Then another CNV is analyzed and its 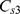 and 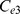 values are compared with the values of the first and second CNVs and the values of the array are modified according the above-described procedure. This procedure is carried out for all CNVs and finally an array with the “start” and “end” values of non-overlapping DNA regions covered by different CNVs is achieved. The total length of CNVs in each chromosome is the sum of the lengths of the corresponding DNA regions. Regarding the mapping of the microRNA genes within the *de novo* CNVs detected in patients, for each chromosome, the programme first initializes a variable to zero and then compares the initial and final nucleotide positions of each CNV with the corresponding nucleotide positions of each miRNA gene. Let us name 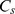, 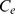, 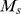, 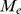 the numeric values corresponding to the first and last nucleotide positions of each CNV and microRNA gene (M), respectively, in a particular chromosome. If the condition 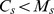 and 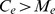 is verified then the count of the number of miRNA genes is increased by one, otherwise the variable remains unchanged. By repeating this procedure for each CNV and each miRNA gene, the total number of microRNA genes overlapping a CNV is obtained for each chromosome.

### Correlation analysis

On the data obtained by the MAPCNVMIR programme (see above) we performed an analysis to evaluate possible correlation between the number of miRNA genes overlapping *de novo* CNVs (“hits”) and the fractional length of CNVs in relation to the size of the chromosome (R_2_ ratios). Briefly, for each chromosome the number of “hits” was plotted against the R_2_ ratios, thus obtaining the linear best fit functions and correlation coefficients were calculated. These analyses were performed separately for CNV_Gains and CNV_Losses.

### SIMCNVMIR program (FORTRAN)

The SIMCNVMIR programme was developed to perform Monte Carlo randomization analyses, separately for CNV_Gains and CNV_Losses in each chromosome. These analyses included: (i) simulation of random CNV events, (ii) generation of a frequency distribution of “hits” in simulated CNVs, (iii) computation of p-values and FDR-adjusted p-values and (iv) selection of chromosomes displaying over-representation of miRNA genes in *de novo* CNVs from patients.

The analysis aimed at evaluating an over-representation of miRNA genes in CNVs was carried out by means of a computational simulation procedure implemented by a home-made written FORTRAN code. The null hypothesis underlying our investigation is that the distribution of CNVs within the chromosome is absolutely random, that is, they can occur anywhere throughout the whole length of a chromosome. Therefore, for each chromosome, the sizes (number of nucleotides) of all CNVs reported in the APL datasets (Table S1) are computed and various realizations of random distributions of these CNV regions within each chromosome are simulated. Once a new distribution of CNVs is obtained, the programme then computes the number of miRNA genes overlapping the simulated CNVs. This procedure was repeated 10^6^ times for each chromosome. Data are then plotted as histograms displaying the occurrence frequency of miRNA genes associated to each CNV. These histograms provide information on the number of times a certain number of miRNAs genes are found to overlap CNVs randomly-distributed in each chromosome. These distributions take into account many factors such as the size of the chromosome, the number and positions of miRNA genes inside the chromosomes, and the size of CNVs. For each chromosome, the number of miRNA genes overall associated to the experimentally observed CNVs (*i.e.*, the CNVs reported in the APL datasets) was compared to the corresponding histogram obtained by the simulation. In order to evaluate whether the number of miRNA genes included in *de novo* CNV in patients is significantly larger than expected with a random distribution of CNVs in each chromosome, we estimated the probability (p-value) of obtaining a number of miRNA genes associated to the simulated CNVs larger than that seen with experimental CNVs. This probability is calculated by summing the area under the histogram for a number of miRNA genes included in CNVs larger than or equal to the experimental value. The p-value is very small if the number of miRNA genes included in experimental CNVs is much larger than the mean value. This means that if the distribution of the CNVs on a given chromosome was random (see the above-mentioned null hypothesis) we would have a low probability of finding a greater number of miRNA genes associated to CNVs. In other words, in autistic patients, CNVs tend to be more frequent in chromosomal regions where the miRNA genes are present than in other regions of the chromosome. The analyses described above have been performed twice: (i) for CNV_Gains and (ii) for CNV_Losses respectively. In our analysis we used a false discovery rate procedure (as multiple hypothesis testing) developed by Benjamini and Hochberg to control the expected proportion of incorrectly rejected null hypotheses [57]. In particular, we exploited a spreadsheet available on-line [58] which calculates FDR-adjusted pvalues from the knowledge of the p-values for the various chromosomes. We set acceptable FDR 0.05 as a maximum (which is the default value of the spreadsheet).

## Supporting Information

Table S1 **The APL dataset of de novo CNVs and the overlapping miRNA genes.**
(PDF)
