# Supplementary material for: Assessing the Impact of Copy Number Variants on miRNA Genes in Autism by Monte Carlo Simulation
Source: PLoS One. 2014 Mar 25;9(3):e90947. doi: 10.1371/journal.pone.0090947 (PMC3965395; doi:10.1371/journal.pone.0090947)
Supplement: Table S1 — The APL dataset of de novo CNVs and the overlapping miRNA genes. (PDF) [file pone.0090947.s001.pdf]

SUPPLEMENTARY INFORMATION

CONTENT

A. LIST OF ABBREVIATIONS **2**

B. TABLE S1 **3**

A. LIST OF ABBREVIATIONS

ACRD, Autism Chromosome Rearrangements Database

APL, ACRD - Pinto et al. 2010 - Levy et al. 2011

CNV, Copy Number Variant

DGV, Database of Genomic Variants

miRNA, microRNA

B. TABLE S1

The APL dataset of *de novo* CNVs and the overlapping miRNA genes.

APL	ID patient	chr	start	end	gain/loss	# miRNA genes	Stem Loop Name
A	103	1	996263	1127268	loss	3	hsa-mir-200b; hsa-mir-200a; hsa-mir-429
A	357	1	97559579	98652079	loss	2	hsa-mir-2682; hsa-mir-137
L	11080_AUT_01	1	31125532	36307613	gain	5	hsa-mir-4420; hsa-mir-4254; hsa-mir-5585; hsa-mir-3605; hsa-mir-552
L	12330_AUT_02	1	240529499	240845702	gain	0	-
L	12330_AUT_03	1	242643393	242910083	gain	0	-
P	13041_503	1	169226902	169240738	loss	0	-
P	5437_3	1	61880686	62134264	gain	0	-
A	129	2	50518349	50873649	loss	0	-
A	130	2	50518349	50873649	loss	0	-
A	323	2	162387213	162486465	loss	0	-
A	324	2	236632455	242985351	loss	7	hsa-mir-4440; hsa-mir-4441; hsa-mir-4269; hsa-mir-2467; hsa-mir-4786; hsa-mir-149; hsa-mir-3133
A	325	2	238435066	242985351	loss	7	hsa-mir-4440; hsa-mir-4441; hsa-mir-4269; hsa-mir-2467; hsa-mir-4786; hsa-mir-149; hsa-mir-3133
A	385	2	186848494	186945624	loss	0	-
L	11152_AUT_04	2	47827304	47842307	loss	0	-
L	11489_AUT_06	2	110734113	111066417	loss	3	hsa-mir-4267; hsa-mir-4436b-1; hsa-mir-4436b-2
L	12119_AUT_05	2	50830144	50873950	loss	0	-
P	13017_223	2	50686373	50877042	loss	0	-
P	13037_463	2	51149072	51304238	loss	0	-
P	13082_963	2	11795138	11823585	gain	0	-
P	13153_1703	2	51136802	51368539	loss	0	-
P	14068_1180	2	50640323	50824331	gain	0	-
P	5089_5	2	102926511	102979028	loss	0	-
P	5451_3	2	179443217	179547860	loss	0	-
A	110	3	185812357	192380293	loss	3	hsa-mir-1248; hsa-mir-28; hsa-mir-944
A	326	3	60770993	60872500	loss	0	-
A	327	3	61097060	61390156	gain	0	-
A	393	3	15150796	16560396	loss	3	hsa-mir-4270; hsa-mir-3134; hsa-mir-563

APL	ID patient	chr	start	end	gain/loss	# miRNA genes	Stem Loop Name
A	398	3	65311260	70550510	loss	2	hsa-mir-4272; hsa-mir-3136
L	11046_AUT_07	3	4158021	4879563	loss	0	-
L	11046_AUT_08	3	5038460	5051532	gain	0	-
L	11079_AUT_16	3	195723966	197354025	loss	1	hsa-mir-4797
L	11223_AUT_13	3	158484738	158500820	loss	0	-
L	11227_AUT_12	3	104969376	105049262	loss	0	-
L	11450_AUT_14	3	164169930	164190404	loss	0	-
L	11479_AUT_11	3	80574659	80606124	loss	0	-
L	11696_AUT_09	3	37277996	37453446	loss	0	-
L	12032_AUT_10	3	67136778	72259085	loss	3	hsa-mir-4272; hsa-mir-3136; hsa-mir-1284
L	12339_AUT_15	3	185612651	185730556	loss	0	-
P	13046_553	3	162521339	162618885	loss	0	-
P	13108_1253	3	162521339	162618885	loss	0	-
P	3174_003	3	19946057	20121828	loss	0	-
P	5220_3	3	19152994	19665295	gain	1	hsa-mir-4791
P	5245_3	3	115802317	115994501	loss	0	-
A	101	4	80282081	82082579	loss	0	-
A	102	4	86288694	101407914	loss	4	hsa-mir-4451; hsa-mir-4452; hsa-mir-5705; hsa-mir-3684
P	13022_293	4	64138341	64150666	loss	0	-
A	429	5	28949	13829933	loss	7	hsa-mir-4456; hsa-mir-4635; hsa-mir-4457; hsa-mir-4277; hsa-mir-4278; hsa-mir-4458; hsa-mir-4636
A	430	5	9222811	12652200	loss	0	-
L	12289_AUT_17	5	11350621	11440124	loss	0	-
A	328	6	13889301	15153952	loss	0	-
P	13007_83	6	812136	849087	gain	0	-
P	13094_1113	6	108308659	108338267	gain	0	-
P	5353_3	6	33291871	33404064	loss	0	-
P	5386_3	6	156743463	158569886	loss	2	hsa-mir-4466; hsa-mir-3692
P	5437_3	6	36332927	36525047	gain	0	-
P	6164_3	6	160103084	160161628	gain	0	-
A	107	7	72379275	74111441	loss	2	hsa-mir-4284; hsa-mir-590
A	330	7	15386878	15538758	loss	0	-
A	464	7	108606430	119629936	loss	1	hsa-mir-3666
A	466	7	113741049	129227770	loss	4	hsa-mir-3666; hsa-mir-592; hsa-mir-593; hsa-mir-129-1

APL	ID patient	chr	start	end	gain/loss	# miRNA genes	Stem Loop Name
A	471	7	154147352	154213814	loss	0	-
L	11129_AUT_19	7	72719808	74143620	gain	2	hsa-mir-4284; hsa-mir-590
L	11154_AUT_20	7	72739232	74142229	gain	2	hsa-mir-4284; hsa-mir-590
L	11868_AUT_18	7	66892296	67101599	gain	0	-
P	1960_301	7	102912596	103011509	loss	0	-
P	5370_3	7	154144653	154213814	loss	0	-
A	472	8	720491	1514173	gain	0	-
L	11039_AUT_22	8	39174221	39182614	loss	0	-
L	11225_AUT_21	8	3977882	4364086	loss	0	-
P	1142_4	8	48468835	48639976	gain	0	-
P	5290_3	8	714383	1534503	gain	0	-
P	6321_3	8	65191812	66092315	loss	1	hsa-mir-124-2
L	11348_AUT_24	9	13301055	13320257	loss	0	-
L	11405_AUT_26	9	95787822	97076025	gain	5	hsa-mir-548au; hsa-mir-4291; has-let-7a-1; hsa-let-7f-1; hsa-let-7d
L	11435_AUT_23	9	202721	3386926	gain	0	-
L	11660_AUT_25	9	30927196	30952603	loss	0	-
L	12235_AUT_27	9	130868096	131472539	gain	4	hsa-mir-199b; hsa-mir-3154; hsa-mir-219-2; hsa-mir-2964a
L	12370_AUT_28	9	134775829	134941327	gain	0	-
L	12581_AUT_29	9	140680187	141020260	loss	1	hsa-mir-602
P	13123_1403	9	108998	3692923	loss	0	-
P	5032_4	9	108998	344508	loss	0	-
P	6246_4	9	9409606	9641169	loss	0	-
A	331	10	50892143	61808505	gain	3	hsa-mir-605; hsa-mir-548f-1; hsa-mir-3924
L	11705_AUT_31	10	68506522	68570019	loss	0	-
L	11718_AUT_32	10	120751118	120981963	gain	0	-
L	11962_AUT_30	10	53027235	54741308	gain	1	hsa-mir-605
L	12100_AUT_33	11	72033899	72045553	loss	0	-
L	12485_AUT_34	11	84553565	84586441	gain	0	-
P	5237_3	11	70476810	70542984	loss	0	-
P	6240_4	11	127128729	132555164	loss	0	-
P	6319_3	11	70442269	70510224	loss	0	-
A	527	12	42297931	42720773	loss	0	-

APL	ID patient	chr	start	end	gain/loss	# miRNA genes	Stem Loop Name
A	537	12	115707280	133777650	gain	10	hsa-mir-620; hsa-mir-4472-2; hsa-mir-1178; hsa-mir-4498; hsa-mir-4700; hsa-mir-4304; hsa-mir-3908; hsa-mir-5188; hsa-mir-4419b; hsa-mir-3612
L	11004_AUT_37	12	122616830	122646647	loss	0	-
L	11714_AUT_35	12	3656361	3667776	loss	0	-
L	12184_AUT_36	12	17016260	29990846	loss	3	hsa-mir-3974; hsa-mir-920; hsa-mir-4302
P	13046_553	12	11515932	11544819	loss	0	-
P	5272_3	12	99921291	100016547	loss	0	-
P	6053_3	12	55932655	60493348	gain	3	hsa-mir-1228; hsa-mir-616; hsa-mir-26a-2
A	329	13	45301441	47245177	loss	0	-
L	11380_AUT_38	13	105763135	105817018	gain	0	-
P	13094_1113	13	64333042	64379372	gain	0	-
P	1050_3	14	21209871	21275334	gain	0	-
A	109	15	20116186	32969479	gain	11	hsa-mir-3118-4; hsa-mir-5701-1; hsa-mir-3118-6; hsa-mir-5701-2; hsa-mir-1268a; hsa-mir-4509-1; hsa-mir-4508; hsa-mir-4715; hsa-mir-4509-2; hsa-mir-4509-3; hsa-mir-211
A	332	15	20167086	32511555	gain	11	hsa-mir-3118-4; hsa-mir-5701-1; hsa-mir-3118-6; hsa-mir-5701-2; hsa-mir-1268a; hsa-mir-4509-1; hsa-mir-4508; hsa-mir-4715; hsa-mir-4509-2; hsa-mir-4509-3; hsa-mir-211
A	558	15	20116186	32511508	gain	11	hsa-mir-3118-4; hsa-mir-5701-1; hsa-mir-3118-6; hsa-mir-5701-2; hsa-mir-1268a; hsa-mir-4509-1; hsa-mir-4508; hsa-mir-4715; hsa-mir-4509-2; hsa-mir-4509-3; hsa-mir-211
A	559	15	20167086	32511555	gain	11	hsa-mir-3118-4; hsa-mir-5701-1; hsa-mir-3118-6; hsa-mir-5701-2; hsa-mir-1268a; hsa-mir-4509-1; hsa-mir-4508; hsa-mir-4715; hsa-mir-4509-2; hsa-mir-4509-3; hsa-mir-211
A	561	15	71814246	76103745	loss	4	hsa-mir-630; hsa-mir-4513; hsa-mir-631; hsa-mir-4313

APL	ID patient	chr	start	end	gain/loss	# miRNA genes	Stem Loop Name
A	652	15	20116186	32969479	gain	11	hsa-mir-3118-4; hsa-mir-5701-1; hsa-mir-3118-6; hsa-mir-5701-2; hsa-mir-1268a; hsa-mir-4509-1; hsa-mir-4508; hsa-mir-4715; hsa-mir-4509-2; hsa-mir-4509-3; hsa-mir-211
A	653	15	20116186	32969479	gain	11	hsa-mir-3118-4; hsa-mir-5701-1; hsa-mir-3118-6; hsa-mir-5701-2; hsa-mir-1268a; hsa-mir-4509-1; hsa-mir-4508; hsa-mir-4715; hsa-mir-4509-2; hsa-mir-4509-3; hsa-mir-211
A	654	15	20116186	32969479	gain	11	hsa-mir-3118-4; hsa-mir-5701-1; hsa-mir-3118-6; hsa-mir-5701-2; hsa-mir-1268a; hsa-mir-4509-1; hsa-mir-4508; hsa-mir-4715; hsa-mir-4509-2; hsa-mir-4509-3; hsa-mir-211
L	11233_AUT_43	15	69099547	74151983	loss	2	hsa-mir-629; hsa-mir-630
L	11265_AUT_40	15	22759123	23088585	loss	0	-
L	11928_AUT_42	15	31076701	32454043	gain	1	hsa-mir-211
L	12007_AUT_39	15	22591745	23070293	gain	1	hsa-mir-4509-1
L	12007_AUT_41	15	23673045	28639666	gain	2	hsa-mir-4508; hsa-mir-4715
P	13050_593	15	23639183	28530359	gain	2	hsa-mir-4508; hsa-mir-4715
P	14070_1230	15	93399003	93482000	loss	1	hsa-mir-3175
P	6101_4	15	76948284	77142762	loss	0	-
A	333	16	6052835	6260815	loss	0	-
A	334	16	29671214	30173788	loss	0	-
A	345	16	29642499	30292499	gain	0	-
A	568	16	29652488	30328317	loss	0	-
A	570	16	29652488	30328317	loss	0	-
A	571	16	29745199	30177799	gain	0	-
A	577	16	89273092	89538759	loss	0	-
A	659	16	29563450	30328317	loss	1	hsa-mir-3680-2
A	660	16	29563450	30328317	loss	1	hsa-mir-3680-2
A	661	16	29563450	30328317	loss	1	hsa-mir-3680-2
A	662	16	29563450	30328317	loss	1	hsa-mir-3680-2
A	663	16	71433886	72324444	gain	0	-
L	11090_AUT_52	16	29651749	30200001	loss	0	-
L	11168_AUT_46	16	8898658	9246454	gain	0	-

APL	ID patient	chr	start	end	gain/loss	# miRNA genes	Stem Loop Name
L	11327_AUT_59	16	81177767	86193522	loss	4	hsa-mir-4720; hsa-mir-3182; hsa-mir-5093; hsa-mir-1910
L	11433_AUT_54	16	29656178	30200001	loss	0	-
L	11435_AUT_44	16	81530	1315850	loss	3	hsa-mir-5587; hsa-mir-3176; hsa-mir-662
L	11511_AUT_47	16	15583359	16383678	gain	1	hsa-mir-484
L	11540_AUT_48	16	29649593	30200001	loss	0	-
L	11551_AUT_45	16	8808569	9302166	gain	0	-
L	11555_AUT_51	16	29651749	30203695	gain	0	-
L	11680_AUT_53	16	29651749	30284160	loss	0	-
L	11800_AUT_60	16	89796985	89809113	loss	0	-
L	12100_AUT_55	16	29665166	30301881	loss	0	-
L	12383_AUT_58	16	77942878	78033587	loss	0	-
L	12435_AUT_50	16	29649593	30200001	gain	0	-
L	12451_AUT_56	16	29665166	30203695	loss	0	-
L	12647_AUT_49	16	29649593	30176674	gain	0	-
L	12736_AUT_57	16	29673618	30205180	gain	0	-
A	338	16	29642499	30292499	loss	0	-
A	339	16	29642499	30292499	loss	0	-
A	340	16	29642499	30292499	loss	0	-
A	341	16	29642499	30292499	loss	0	-
A	342	16	29642499	30292499	loss	0	-
A	346	16	29642499	30292499	loss	0	-
A	347	16	29642499	30292499	loss	0	-
A	348	16	29642499	30292499	loss	0	-
A	350	16	29642499	30292499	loss	0	-
A	351	16	29642499	30292499	loss	0	-
P	5068_3	16	29595483	30219525	loss	1	hsa-mir-3680-2
P	5262_4	16	29595483	30303348	gain	1	hsa-mir-3680-2
P	5359_4	16	29647342	30287723	loss	0	-
P	5437_3	16	16630813	18361376	loss	0	-
L	11186_AUT_65	17	79017703	79066515	loss	0	-
L	11353_AUT_62	17	34734524	36221700	loss	1	hsa-mir-2909
L	11454_AUT_63	17	43686404	44168013	loss	0	-
L	11532_AUT_61	17	6091605	7353817	gain	5	hsa-mir-4520a; hsa-mir-4520b; hsa-mir-195; hsa-mir-497; hsa-mir-324
L	11982_AUT_64	17	44780004	45121631	gain	1	hsa-mir-5089
P	5056_4	17	37358682	37478801	gain	0	-

APL	ID patient	chr	start	end	gain/loss	# miRNA genes	Stem Loop Name
P	5444_3	17	80192650	80256428	loss	0	-
P	5444_3	17	79338469	80188978	gain	2	hsa-mir-4740; hsa-mir-3186
A	588	18	57605621	78014628	loss	2	hsa-mir-5011; hsa-mir-548av
L	11705_AUT_66	18	49917089	49960765	gain	0	-
L	12521_AUT_67	18	55457265	55486887	loss	0	-
L	11937_AUT_68	19	37677894	37796685	loss	0	-
P	6358_6	19	4597413	5336389	loss	2	hsa-mir-7-3; hsa-mir-4747
A	335	20	127912	419871	loss	0	-
A	336	20	2837194	4006399	loss	2	hsa-mir-103a-2; hsa-mir-103b-2
P	5046_3	20	8659242	8689441	loss	0	-
P	5335_3	20	14597734	15000785	loss	0	-
A	104	21	39154938	44847089	loss	3	hsa-mir-4760; hsa-mir-3197; hsa-mir-5692b
A	603	21	44101079	44455015	gain	1	hsa-mir-5692b
A	337	22	46823508	51175739	loss	3	hsa-mir-3201; hsa-mir-4535; hsa-mir-3667
A	605	22	18640346	21461646	loss	6	hsa-mir-4761; hsa-mir-185; hsa-mir-3618; hsa-mir-1306; hsa-mir-1286; hsa-mir-649
A	607	22	18890946	23222208	gain	9	hsa-mir-4761; hsa-mir-185; hsa-mir-3618; hsa-mir-1306; hsa-mir-1286; hsa-mir-649; hsa-mir-301b; hsa-mir-130b; hsa-mir-650
A	609	22	47956881	51218956	loss	3	hsa-mir-3201; hsa-mir-4535; hsa-mir-3667
A	612	22	50953103	51229805	loss	0	-
A	674	22	18640346	23222208	gain	9	hsa-mir-4761; hsa-mir-185; hsa-mir-3618; hsa-mir-1306; hsa-mir-1286; hsa-mir-649; hsa-mir-301b; hsa-mir-130b; hsa-mir-650
L	11723_AUT_71	22	28587065	28611353	loss	0	-
L	12224_AUT_72	22	40495436	40771063	loss	0	-
L	12239_AUT_69	22	18869508	20313261	loss	5	hsa-mir-4761; hsa-mir-185; hsa-mir-3618; hsa-mir-1306; hsa-mir-1286
L	12239_AUT_70	22	20707910	21465854	loss	1	hsa-mir-649

APL	ID patient	chr	start	end	gain/loss	# miRNA genes	Stem Loop Name
P	3183_7	22	18861748	21489918	loss	6	hsa-mir-4761; hsa-mir-185; hsa-mir-3618; hsa-mir-1306; hsa-mir-1286; hsa-mir-649
A	613	X	94419	5999994	loss	1	hsa-mir-3690
A	618	X	48317346	52833945	gain	8	hsa-mir-532; hsa-mir-188; hsa-mir-500a; hsa-mir-362; hsa-mir-501; hsa-mir-500b; hsa-mir-660; hsa-mir-502
L	11092_AUT_75	X	153576690	153779907	gain	0	-
L	11689_AUT_74	X	70379448	70413264	loss	0	-
L	12561_AUT_73	X	22944530	23302214	loss	0	-

APL (source of data): A = Autism Chromosome Rearrangement Database[1], P = Pinto et al.[2]; L=Levy et al.[3]; start/end: Genomic coordinates are according to HG19; # miRNA genes/Stem Loop Name: number and Stem Loop Name of miRNA genes overlapping each *de novo* CNV were computed and mapped by the MAPCNVMIR program (see "Methods" section).
